# Vitamin D Supplementation Associated to Better Survival in Hospitalized Frail Elderly COVID-19 Patients: The GERIA-COVID Quasi-Experimental Study

**DOI:** 10.3390/nu12113377

**Published:** 2020-11-02

**Authors:** Gaëlle Annweiler, Mathieu Corvaisier, Jennifer Gautier, Vincent Dubée, Erick Legrand, Guillaume Sacco, Cédric Annweiler

**Affiliations:** 1 School of Medicine, Health Faculty, University of Angers, 49045 Angers, France; gaelle.annweiler@gmail.com (G.A.); Vincent.Dubee@chu-angers.fr (V.D.); ErLegrand@chu-angers.fr (E.L.); 2 Department of Medicine, Clinique de l’Anjou, 49044 Angers, France; 3 Department of Geriatric Medicine, Research Center on Autonomy and Longevity, University Hospital, 49933 Angers, France; Mathieu.Corvaisier@chu-angers.fr (M.C.); JeGautier@chu-angers.fr (J.G.); yogisacco@gmail.com (G.S.); 4 Department of Pharmacy, Angers University Hospital, 49933 Angers, France; 5 Nantes-Angers Cancer and Immunology Research Center (CRCINA), Inserm, University of Angers, 49000 Angers, France; 6 Department of Infectious and Tropical Diseases, Angers University Hospital, 49933 Angers, France; 7 Department of Rheumatology, Angers University Hospital, 49933 Angers, France; 8 EA4638, Laboratory of Psychology of the Pays de la Loire, University of Angers, 49045 Angers, France; 9 Gérontopôle of Pays de la Loire, 44000 Nantes, France; 10 Robarts Research Institute, Department of Medical Biophysics, Schulich School of Medicine and Dentistry, the University of Western Ontario, London, ON N6A 5K8, Canada

**Keywords:** COVID-19, SARS-CoV-2, vitamin D, therapeutics, quasi-experimental study, older adults

## Abstract

Background. The objective of this quasi-experimental study was to determine whether bolus vitamin D supplementation taken either regularly over the preceding year or after the diagnosis of COVID-19 was effective in improving survival among hospitalized frail elderly COVID-19 patients. Methods. Seventy-seven patients consecutively hospitalized for COVID-19 in a geriatric unit were included. Intervention groups were participants regularly supplemented with vitamin D over the preceding year (Group 1), and those supplemented with vitamin D after COVID-19 diagnosis (Group 2). The comparator group involved participants having received no vitamin D supplements (Group 3). Outcomes were 14-day mortality and highest (worst) score on the ordinal scale for clinical improvement (OSCI) measured during COVID-19 acute phase. Potential confounders were age, gender, functional abilities, undernutrition, cancer, hypertension, cardiomyopathy, glycated hemoglobin, number of acute health issues at admission, hospital use of antibiotics, corticosteroids, and pharmacological treatments of respiratory disorders. Results. The three groups (*n* = 77; mean ± SD, 88 ± 5 years; 49% women) were similar at baseline (except for woman proportion, *p* = 0.02), as were the treatments used for COVID-19. In Group 1 (*n* = 29), 93.1% of COVID-19 participants survived at day 14, compared to 81.2% survivors in Group 2 (*n* = 16) (*p* = 0.33) and 68.7% survivors in Group 3 (*n* = 32) (*p* = 0.02). While considering Group 3 as reference (hazard ratio (HR) = 1), the fully-adjusted HR for 14-day mortality was HR = 0.07 (*p* = 0.017) for Group 1 and HR = 0.37 (*p* = 0.28) for Group 2. Group 1 had longer survival time than Group 3 (log-rank *p* = 0.015), although there was no difference between Groups 2 and 3 (log-rank *p* = 0.32). Group 1, but not Group 2 (*p* = 0.40), was associated with lower risk of OSCI score ≥5 compared to Group 3 (odds ratio = 0.08, *p* = 0.03). Conclusions. Regular bolus vitamin D supplementation was associated with less severe COVID-19 and better survival in frail elderly.

## 1. Introduction

Since December 2019, the COVID-19 caused by SARS-CoV-2 is spreading worldwide, affecting millions of people and leaving hundreds of thousands dead, mostly in older adults. With the lack of effective therapy, chemoprevention, and vaccination [1], focusing on the immediate repurposing of existing drugs gives hope of curbing the pandemic. Importantly, a recent unbiased genomics-guided tracing of the SARS-CoV-2 targets in human cells identified vitamin D among the three top-scoring molecules manifesting potential infection mitigation patterns through their effects on gene expression [2]. In particular, by activating or repressing several genes in the promoter region of which it binds to the vitamin D response element, [3] vitamin D may theoretically prevent or improve COVID-19 adverse outcomes by regulating i) the renin–angiotensin system (RAS), ii) the innate and adaptive cellular immunity, iii) the physical barriers, and iv) the host frailty and comorbidities [4,5]. Consistently, epidemiology shows that hypovitaminosis D is more common from October to March at northern latitudes above 20 degrees, [3] which corresponds to the latitudes with the highest lethality rates of COVID-19 during the first months of winter 2020 [1]. In line with this, significant inverse associations were found in European countries between serum 25-hydroxyvitamin D (25(OH)D) concentration and the number of COVID-19 cases, as well as with COVID-19 mortality [6]. This suggests that increasing serum 25(OH)D concentration may improve the prognosis of COVID-19. However, no large well-designed randomized controlled trial (RCT) has tested the effect of vitamin D supplements on COVID-19 outcomes yet. We had the opportunity to examine the association between the use of bolus vitamin D supplements and COVID-19 outcomes in a sample of hospitalized frail elderly patients infected with SARS-CoV-2. The main objective of this hospital-based quasi-experimental study was to determine whether bolus vitamin D supplementation taken either regularly during the preceding year or after the diagnosis of COVID-19 was effective in improving survival among frail elderly COVID-19 patients. The secondary objective was to determine whether this intervention was also effective in limiting the clinical severity of the infection.

## 2. Materials and Methods

### 2.1. Study Population

The study consisted in a quasi-experimental study conducted in one geriatric acute care unit dedicated to COVID-19 patients. Data of the GERIA-COVID study were retrospectively collected from patients’ records. The inclusion criteria were as follows: (1) patients hospitalized in the geriatric acute care unit of Angers University Hospital, France, in March–May 2020; (2) no objection from the patient and/or relatives to the use of anonymized clinical and biological data for research purpose. The inclusion criteria for the present analysis were as follows: (1) COVID-19 diagnosed with RT-PCR and/or chest CT-scan; (2) data available on the treatments received, including vitamin D supplementation, since the diagnosis of COVID-19 and over the preceding year at least; (3) data available on the vital status 14 days after the diagnosis of COVID-19. Seventy-seven patients were consecutively diagnosed with COVID-19 during the study period in the unit. All of them were recruited in the GERIA-COVID study. They all met the other inclusion criteria and were included in the present analysis.

### 2.2. Intervention: Vitamin D Supplementation

The regular intake of bolus vitamin D supplements over the preceding year was systematically noted from the primary care physicians’ prescriptions and sought by questioning the patients and their relatives. 

“Group 1” was defined as all COVID-19 patients who had received oral boluses of vitamin D supplements over the preceding year. Bolus included the doses of 50,000 IU vitamin D3 per month, or the doses of 80,000 IU or 100,000 IU vitamin D3 every 2–3 months. None received D2 or intramuscular supplements, and no patient in Group 1 received additional supplements following the diagnosis of COVID-19.

“Group 2” was defined as the COVID-19 patients usually not supplemented with vitamin D, but who received an oral supplement of 80,000 IU vitamin D3 within a few hours of the diagnosis of COVID-19.

Finally, “Group 3” was defined as the Comparator group, i.e., all COVID-19 patients who had received no vitamin D supplements, neither over the preceding year nor after the diagnosis of COVID-19; the absence of vitamin D treatment being mostly explained by the patients’ refusal to be supplemented, since vitamin D supplementation is recommended with no biological testing in all patients over 65 years of age in France [3].

### 2.3. Primary Outcome: 14-Day COVID-19 Mortality

The primary outcome was the 14-day mortality. Follow-up started from the day of COVID-19 diagnosis for each patient and continued for 14 days or until death when applicable.

### 2.4. Secondary Outcome: Ordinal Scale for Clinical Improvement (OSCI) Score for COVID-19 in Acute Phase 

The secondary outcome was the score on the 9-point World Health Organization’s ordinal scale for clinical improvement (OSCI) for COVID-19 [7]. The OSCI distinguishes between several levels of COVID-19 clinical severity according to the outcomes and dedicated treatments required, with a score ranging from 0 (no clinical or virological sign of infection) to 8 (death). The score was determined by the geriatrician of the hospital unit on admission, then revised regularly according to the clinical course of the patients. The highest score during hospitalization was used for the present analysis, corresponding to the most severe acute phase of COVID-19 for each patient. A score of 3 corresponds to a degree of severity requiring hospitalization (i.e., all GERIA-COVID participants had an OSCI score ≥3 here), a score of 5 corresponds to the introduction of non-invasive ventilation, and a score of 6 to intubation and invasive ventilation [7]. Severe COVID-19 was defined here as a score of 5 or more.

### 2.5. Covariables

Potential confounders were age, gender, functional abilities, severe undernutrition, history of cancer, hypertension, cardiomyopathy, glycated hemoglobin, number of acute health issues at admission, hospital use of antibiotics, systemic corticosteroids, and pharmacological treatments of respiratory disorders. Functional abilities prior to COVID-19 were measured from 1 to 6 (best) with the iso-resources groups (GIR) [8]. Serum albumin concentration, C-reactive protein (CRP), and glycated hemoglobin were measured at hospital admission. Severe undernutrition was defined as albumin <30 g/L. Acute health issues were defined as diseases with sudden onset and rapid progression, whatever their nature or site [9]. History of hematological and solid cancers, hypertension, and cardiomyopathy were noted from the medical register, and by interviewing patients, their relatives, and family physicians. The use of systemic corticosteroids and/or antibiotics (i.e., quinolones, beta-lactams, sulfonamides, macrolides, lincosamides, aminoglycosides, among others), and/or pharmacological treatments of respiratory disorders (i.e., beta2-adrenergic agonists, inhaled corticosteroids, antihistamines, among others) were noted from prescriptions during hospitalization.

### 2.6. Statistical Analysis

The participants’ characteristics were summarized using means and standard deviations (SD) or frequencies and percentages, as appropriate. As the number of observations was higher than 40, comparisons were not affected by the shape of the error distribution and no transformation was applied [10]. Firstly, comparisons between participants separated into three groups according to the intervention (i.e., regular supplementation versus supplementation initiated after COVID-19 diagnosis versus no supplementation) were performed using analysis of variance (ANOVA) or Mann–Whitney–U and Kruskal–Wallis tests for quantitative variables as appropriate, and using Chi-square test or Fisher exact test for qualitative variables as appropriate. To address the issue of multiple comparisons, analyses were completed by a post hoc Fisher’s least significant difference (LSD) test. Secondly, a fully-adjusted Cox regression was used to examine the associations of 14-day mortality (dependent variable) with vitamin D supplementation and covariables (independent variables). The model produces a survival function that provides the probability of death at a given time for the characteristics supplied for the independent variables. Third, the elapsed time to death was studied by survival curves computed according to the Kaplan–Meier method and compared by log-rank test. Finally, a multiple logistic regression was used to examine the association of vitamin D supplementation (independent variable) with severe COVID-19 defined as an OSCI score ≥5 (dependent variable), while adjusting for potential confounders. *p*-values <0.05 were considered significant. All statistics were performed using SPSS (v23.0, IBM Corporation, Chicago, IL, USA) and SAS (v9.4, Sas Institute Inc, Cary, NC, USA).

### 2.7. Ethics

The study was conducted in accordance with the ethical standards set forth in the Helsinki Declaration (1983). No participant or relatives objected to the use of anonymized clinical and biological data for research purposes. Ethics approval was obtained from the Ethics Board of the University Hospital of Angers, France (2020/100). The study protocol was also declared to the National Commission for Information Technology and civil Liberties (CNIL; ar20-0087v0) and registered on ClinicalTrials.gov (NCT04560608).

## 3. Results

Seventy-seven participants (mean ± SD age 88 ± 5 years, range 78−100 years; 49.4% women) were included in this quasi-experimental study. Seventeen participants experienced severe COVID-19, and 62 participants survived COVID-19 at day 14, while 15 died.

Table 1 indicates the characteristics of participants separated into Group 1 who regularly received vitamin D supplements over the preceding year (*n* = 29), Group 2 who received vitamin D supplements after the diagnosis of COVID-19 (*n* = 16), and Group 3 who had not received vitamin D supplements (*n* = 32). The three groups were similar at baseline with no significant difference regarding the age (*p* = 0.22), the functional abilities (*p* = 0.36), the history of various comorbidities, the number of acute health issues at hospital admission (*p* = 0.22), and the use of treatments dedicated to COVID-19 (Table 1). At hospital admission, all participants had an OSCI score for COVID-19 of 3 or more. Only the proportion of women differed between groups (*p* = 0.02). At the end of the study, the proportion of participants experiencing severe COVID-19 was lower in Group 1 (10.3%) compared to Group 3 (31.3%, *p* = 0.047), just like the 14-day mortality (6.9% in Group 1 versus 31.3% in Group 3, *p* = 0.02). In contrast, participants in Group 2 did not experience less severe COVID-19 (*p* = 0.75) and less mortality (*p* = 0.50) than participants in Group 3 (Table 1). Similarly, there were no outcome differences between Groups 1 and 2 (*p* = 0.23 for the onset of severe COVID-19, and *p* = 0.33 for 14-day mortality).

**Figure 1:**
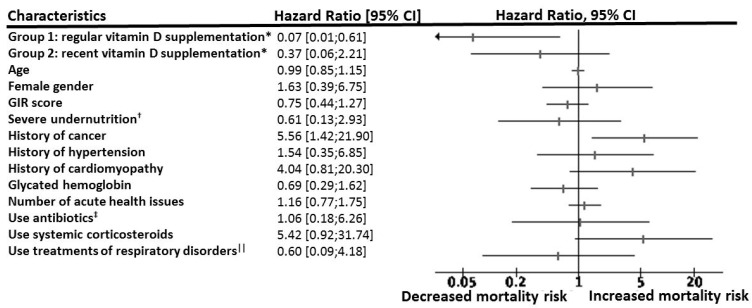
Hazard ratio for 14-day mortality according to vitamin D interventions among participants with COVID-19, adjusted for potential confounders (*n* = 77). CI: confidence interval; COVID-19: coronavirus disease 2019; GIR: Iso Resource Groups; HR: hazard ratio; OSCI: World Health Organization’s Ordinal Scale for Clinical Improvement; *: while using Group 3 (no vitamin D supplementation) as a reference (HR = 1); ^†^: serum albumin concentration <30 g/L; ^‡^: quinolones, beta-lactams, sulfonamides, macrolides, lincosamides, aminoglycosides, among others; ^||^: beta2-adrenergic agonists, inhaled corticosteroids, antihistamines, among others.

**Figure 2:**
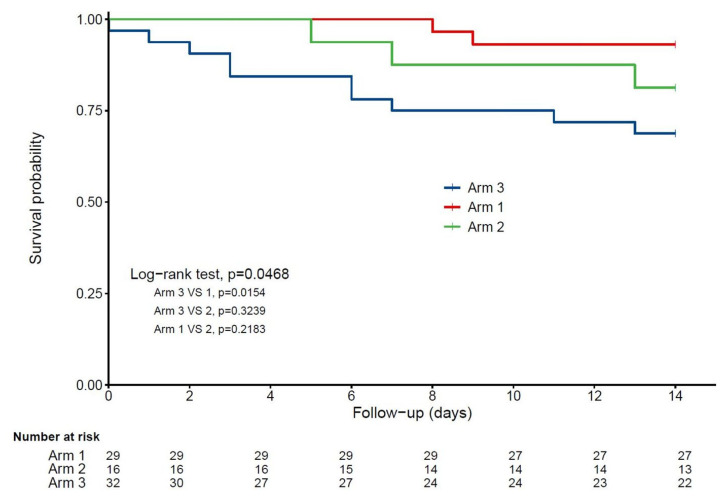
Kaplan–Meier estimates of the cumulative probability of COVID-19 participants’ survival according to vitamin D interventions (*n* = 77). Arm 1: regular vitamin D supplementation; Arm 2: vitamin D supplementation initiated after COVID-19 diagnosis; Arm 3: no vitamin D supplementation.

Figure 1 shows a statistically significant and clinically relevant inverse association between regular vitamin D supplementation and 14-day mortality. While considering Group 3 as the reference (hazard ratio (HR) = 1), the HR for mortality in Group 1 was 0.19 (95% confidence interval (95% CI): 0.04; 0.85) (*p* = 0.03) in the unadjusted model, HR = 0.18 (95% CI: 0.04; 0.85) (*p* = 0.03) after partial adjustment for age, gender and GIR score, and HR = 0.07 (95% CI: 0.01; 0.61) (*p* = 0.017) after full adjustment for all potential confounders. In contrast, being supplemented with vitamin D after the diagnosis of COVID-19 (Group 2) was not associated with lower mortality risk (HR = 0.37 (95% CI): 0.06; 2.21), *p* = 0.28). The history of hematological and solid cancers was associated with greater mortality risk (HR = 5.56, *p* = 0.01). Using the season of COVID-19 diagnosis as an additional potential confounder did not affect the results (data not shown). Consistently, Kaplan–Meier distributions showed in Figure 2 that COVID-19 participants in Group 3 had shorter survival time than those in Group 1 (log-rank *p* = 0.015), although there was no difference between Groups 2 and 3 (log-rank *p* = 0.32) and between Groups 1 and 2 (log-rank *p* = 0.22).

Finally, the multiple logistic regression model in Table 2 revealed that regular vitamin D supplementation (Group 1) was associated with a lower proportion of participants with severe COVID-19 in acute phase (odds ratio (OR) = 0.08 (95% CI): 0.01; 0.81), *p* = 0.033) compared to Group 3 without vitamin D supplementation. In contrast, Group 2 was not associated with any beneficial effect compared to Group 3 (OR = 0.46 (95% CI): 0.07; 2.85), *p* = 0.40).

## 4. Discussion

The main finding of this quasi-experimental study is that, irrespective of all measured potential confounders, regular bolus vitamin D3 supplementation was associated with less severe COVID-19 and better survival rate in hospitalized frail elderly. Being supplemented with 80,000 IU vitamin D3 after the diagnosis of COVID-19 was not associated with improved COVID-19 outcomes. These novel findings provide a scientific basis for vitamin D replacement trials attempting to improve COVID-19 prognosis.

To our knowledge, we provide here the first quasi-experimental data comparing the effects of chronic and recent vitamin D supplementations on survival in COVID-19 patients. Growing evidence supports a link between vitamin D and COVID-19. The first reports indicated that adults with hypovitaminosis D were at greater risk of being infected with SARS-CoV-2 (relative risk 1.77 with *p* < 0.02), [11] and that cases with COVID-19 had lower 25(OH)D concentrations compared to controls without COVID-19 (respectively, 11.1 ng/mL versus 24.6 ng/mL, *p* = 0.004) [12]. Similarly, significant inverse correlations were found in 20 European countries between the mean serum 25(OH)D concentrations and the number of COVID-19 cases, as well as with mortality [6]. The severity of hypovitaminosis D appears to relate to the prognosis of COVID-19, since COVID-19 cases with hypovitaminosis D were more prone to experience severe COVID-19 (relative risk 1.59 with *p* = 0.02 if vitamin D insufficiency <30ng/mL) [13]. Finally, hypovitaminosis D was found to be associated with greater COVID-19 mortality risk (incident relative risk 1.56 with *p* < 0.001 if vitamin D deficiency; *p* = 0.404 after adjustment) [14]. These results support that enhancing serum 25(OH)D concentration may improve the prognosis of COVID-19, as demonstrated by a pilot controlled trial reporting that the administration of calcifediol versus no calcifediol reduced the need for ICU treatment in 76 hospitalized participants with COVID-19 also receiving best available therapy (mean age, 53 years; 40.8% women) [15]. Following these preliminary findings, larger interventional studies dedicated to COVID-19 with groups properly matched are warranted for investigating the role of vitamin D supplementation on COVID-19 outcomes. Interestingly, previous meta-analyses found that high-dose prophylactic vitamin D supplementation was able to reduce the risk of respiratory tract infections [16]. Based on this observation, we and others are conducting an RCT, the COVIT-TRIAL study, designed to test the effect of high-dose versus standard-dose vitamin D3 on 14-day mortality in COVID-19 older patients (https://clinicaltrials.gov/ct2/show/NCT04344041). While waiting for the recruitment of this RCT to be completed, the findings of the present quasi-experimental study strongly suggest benefits of regular vitamin D3 supplementation on COVID-19 outcomes and survival, which reinforces the recommendations of some scientific societies to supplement all elderly people with vitamin D, in order to improve COVID-19 mortality [17,18]. Additionally, our results support the observation that a single standard dose of 80,000 IU vitamin D3 initiated after the diagnosis of COVID-19 brings no significant benefit on COVID-19 outcomes, which justifies using low-dose vitamin D supplements as a comparator in the COVIT-TRIAL study to determine the effect of higher-dose vitamin D supplements on the prognosis of COVID-19.

How vitamin D supplementation improves COVID-19 outcomes and survival is not fully elucidated. Four mechanisms are likely: regulation of i) the RAS, ii) the innate and adaptive cellular immunity, iii) the physical barriers, and iv) the host frailty and comorbidities [3,4,5]. First, vitamin D reduces pulmonary permeability in animal models of acute respiratory distress syndrome (ARDS) by modulating the activity of RAS and the expression of the angiotensin-2 converting enzyme (ACE2) [19]. This action is crucial since SARS-CoV-2 reportedly uses ACE2 as a receptor to infect host cells [20] and downregulates ACE2 expression [21]. ACE2 is expressed in many organs, including the endothelium and the pulmonary alveolar epithelial cells, where it has protective effects against inflammation [22]. During COVID-19, downregulation of ACE2 results in an inflammatory chain reaction, the cytokine storm, complicated by ARDS [23]. In contrast, a study in rats with chemically-induced ARDS showed that the administration of vitamin D increased the levels of ACE2 mRNA and proteins [24]. Rats supplemented with vitamin D had milder ARDS symptoms and moderate lung damage compared to controls. Second, many studies have described the antiviral effects of vitamin D, which works either by induction of antimicrobial peptides with direct antiviral activity against enveloped and non-enveloped viruses, or by immunomodulatory and anti-inflammatory effects [25]. These are potentially important during COVID-19 to limit the cytokine storm. Vitamin D can prevent ARDS [26] by reducing the production of pro-inflammatory Th1 cytokines, such as TNFα and interferon γ [26]. It also increases the expression of anti-inflammatory cytokines by macrophages [25]. Third, vitamin D stabilizes physical barriers [4]. These barriers are made up of closely linked cells to prevent outside agents (such as viruses) from reaching tissues susceptible to viral infection. Although viruses alter the integrity of the cell junction, vitamin D contributes to the maintenance of functional tight junctions via E-cadherin [4]. Fourth, the literature over the past decade on the non-bone effects of vitamin D has repeatedly reported that hypovitaminosis D is accompanied by various comorbidities including diabetes mellitus, hypertension, chronic cardiovascular and respiratory diseases, and cancers [3], all conditions that are associated with an increased risk of COVID-19 worsening and death [1]. Prolonged hypovitaminosis D may thus be considered as a factor of poor prognosis of COVID-19, potentiating the risk of cardiorespiratory severity in frail older adults infected with SARS-CoV-2.

All these actions of vitamin D may explain the protective effect of regular long-term vitamin D supplementation, the latter providing the body with a desirable vitamin D environment allowing the various beneficial effects to be expressed and potentiated in the protection against COVID-19. On the contrary, we assume that vitamin D supplementation initiated after the diagnosis of COVID-19 was started too late for the effects of vitamin D to be effective against the infection. It is also possible that the single dose of 80,000 IU was too low to generate protective effects in a very short time, a hypothesis tested in the COVIT-TRIAL RCT.

We also noted, in the present study, a 14-day mortality rate of 31.3% among frail older adults not supplemented with vitamin D (Table 1). This result is consistent with previous literature that points out a special vulnerability of frail older adults. Mortality is less than 1.1% in patients aged <50 years and it increases exponentially after that age up to around 30% [27], especially in frail older adults who have the highest proportion of severe cases of COVID-19 and fatal outcomes [28]. Thus, this result validates the consistency of our cohort and of our main results, notably the protective effect of the regular intake of vitamin D supplements on COVID-19 outcomes.

The strengths of the present study include (i) the originality of the research question on an emerging infection for which there is no scientifically validated treatment [1], (ii) the follow-up and the detailed description of the participants’ characteristics allowing the use of multivariate Cox models to measure adjusted longitudinal associations according to three vitamin D regimens, and (iii) the standardized collection of data from a single research center.

Regardless of that, a number of limitations also existed. First, the study participants were restricted to a limited number of hospitalized frail elderly patients who might be unrepresentative of all older adults. It is also possible that the limited sample size in each group had resulted in a lack of power with increased beta risk. Second, although we were able to control for the important characteristics that could modify the association, residual potential confounders might still be present such as the serum concentration of 25(OH)D at baseline—a low level classically ensuring the efficacy of the supplementation [29], or the OSCI score on admission. The OSCI score was collected here in the most acute phase of COVID-19 as it was reported that COVID-19 can get worse between 7–10 days due to the cytokine storm regardless of the initial disease severity [30]. Third, the quasi-experimental design of our study is less robust than an RCT. Participants in the Comparator group did not receive vitamin D placebo. Moreover, there was no randomization. It is plausible that the participants who regularly received vitamin D supplementation (Group 1) were treated better by their family physicians than the others, thereby exhibiting more stable chronic diseases such as cardiovascular comorbidities. It is also plausible that patients or relatives refused taking vitamin D supplementation in Group 3, because the conditions of patients were too severe for them to take the supplements. It should yet be noted that the history did not differ between the 3 groups and that their demographical and health characteristics were similar at baseline, except for the proportion of women (who are likely to suffer from osteoporosis and may have received corresponding treatment that includes vitamin D). While gender is a recognized prognostic factor for COVID-19 [30], the effect of vitamin D supplementation on COVID-19 outcomes persisted after adjustment for all studied confounders including the gender, which allows interpreting the severity and survival differences as being explained by the interventions based on vitamin D supplementation.

## 5. Conclusions 

In conclusion, we were able to report, among hospitalized frail elderly patients with COVID-19, that regular bolus vitamin D3 supplementation was associated with less severe COVID-19 and better survival rate. Vitamin D3 supplementation may represent an effective, accessible, and well-tolerated adjuvant treatment for COVID-19, the incidence of which increases dramatically and for which there are currently no validated treatments. Further large prospective, preferentially interventional studies are needed to confirm whether supplementing older adults regularly with vitamin D3 prevents COVID-19 onset and/or improves COVID-19 outcomes; and whether higher-dose bolus of vitamin D3 given after the diagnosis of COVID-19 is able to improve its prognosis.

## Figures and Tables

**Table 1:** Characteristics and comparisons of participants with COVID-19 according to the study groups (*n* = 77).

	All COVID-19 Participants (*n* = 77)	Study Groups			*p*-Value *			
		Group 1 Regular Vitamin D Supplementation (*n* = 29)	Group 2 Vitamin D Supplementation After COVID-19 Diagnosis (*n* = 16)	Group 3 Non-Supplemented Comparator Group (*n* = 32)	Overall (*n* = 77)	Group 1 Versus Group 3 (*n* = 61)	Group 2 Versus Group 3 (*n* = 48)	Group 1 Versus Group 2 (*n* = 45)
**Demographical data**								
Age (years), med (IQR)	88 (85–92)	88 (87–93)	85 (84–89)	88 (84–92)	0.22	0.98	0.12	0.10
Female gender	38 (49.4)	20 (69.0)	5(31.3)	13 (40.6)	**0.02**	**0.027**	0.52	**0.015**
GIR score (/6), med (IQR)	4 (2–4)	4 (3–4)	4 (3–5)	4 (2–5)	0.36	0.63	0.34	0.13
**Comorbidities**								
Severe undernutrition ^†^	21 (27.3)	9 (31.0)	3 (18.8)	9 (28.1)	0.67	0.80	0.73	0.49
Hematological and solid cancers	27 (35.1)	10 (34.5)	4 (25.0)	13 (40.6)	0.56	0.62	0.29	0.74
Hypertension	49 (63.6)	18 (62.1)	10 (62.5)	21 (65.6)	0.95	0.77	0.83	0.98
Cardiomyopathy	42 (54.5)	13 (44.8)	11 (68.8)	18 (56.3)	0.30	0.37	0.40	0.12
Glycated hemoglobin (%), med (IQR)	6.2 (5.8–6.7)	6.0 (5.5–6.6)	6.4 (6.0–8.2)	6.2 (5.9–6.7)	0.16	0.19	0.34	0.08
**Hospitalization**								
Number of acute health issues at hospital admission, med (IQR)	3.0 (2.0–4.0)	3.0 (2.0–4.0)	3.5 (2.0–5.0)	2.5 (1.0–4.0)	0.22	0.18	0.14	0.62
CRP at admission (mg/L), med (IQR)	59.5 (19.5–135.0)	44.0 (19.0–110.0)	69.0 (15.5–140.0)	59.0 (29.0–166.0)	0.47	0.21	0.67	0.63
Use of antibiotics ^‡^	59 (76.6)	23 (79.3)	14 (87.5)	22 (68.8)	0.32	0.349	0.29	0.69
Use of systemic corticosteroids	13 (16.9)	6 (20.7)	2 (12.5)	5 (15.6)	0.79	0.607	1.00	0.69
Use of pharmacological treatments of respiratory disorders ^||^	10 (13.0)	1 (3.5)	2 (12.5)	7 (21.9)	0.10	0.055	0.70	0.29
**COVID-19 outcomes**								
Severe COVID-19 ^§^	17 (22.1)	3 (10.3)	4 (25.0)	10 (31.3)	0.14	**0.047**	0.75	0.23
14-day mortality	15 (19.5)	2 (6.9)	3 (18.8)	10 (31.3)	0.06	**0.017**	0.50	0.33

Data presented as *n* (%) where applicable; COVID-19: Coronavirus Disease 2019; CRP: C-reactive protein; GIR: Iso Resource Groups; IQR: interquartile range; OSCI: Ordinal Scale for Clinical Improvement of the World Health Organization; *: between-group comparisons based on Chi-square test (or Fisher exact test where applicable) and ANOVA (or Mann–Whitney–U or Kruskal–Wallis test where applicable); ^†^: serum albumin concentration <30 g/L; ^‡^: quinolones, beta-lactams, sulfonamides, macrolides, lincosamides, aminoglycosides, among others; ^||^: beta2-adrenergic agonists, inhaled corticosteroids, antihistamines, among others; ^§^: defined as an OSCI score for COVID-19 in acute phase ≥5.

**Table 2:** Multiple logistic regressions showing the association between vitamin D interventions (independent variable) and the risk of severe COVID-19 * (dependent variable), adjusted for participants’ characteristics (*n* = 77).

	Severe COVID-19 *		
	OR	(95% CI)	*p*-Value
Interventions			
Group 1: regular vitamin D supplementation	0.08	(0.01; 0.81)	**0.033**
Group 2: vitamin D supplementation initiated after COVID-19 diagnosis	0.46	(0.07; 2.85)	0.40
Group 3: no vitamin D supplementation	1		
Age	1.05	(0.88; 1.25)	0.61
Female gender	1.43	(1.29; 7.13)	0.66
GIR score	0.76	(0.44; 1.33)	0.33
Severe undernutrition ^†^	0.42	(0.07; 2.48)	0.34
History of cancer	7.30	(1.37; 38.8)	**0.02**
History of hypertension	0.51	(0.11; 2.33)	0.39
History of cardiomyopathy	10.01	(1.44; 69.88)	**0.02**
Glycated hemoglobin ^‡^	0.96	(0.56; 1.63)	0.87
Number of acute health issues at hospital admission	1.19	(0.76; 1.88)	0.45
Use of antibiotics ^||^	1.12	(0.18; 6.85)	0.91
Use of systemic corticosteroids	2.53	(0.34; 17.00)	0.34
Use of pharmacological treatments of respiratory disorders ^§^	0.26	(0.02; 2.86)	0.27

CI: confidence interval; COVID-19: coronavirus disease 2019; GIR: Iso Resource Groups; OR: odds ratio; OSCI: World Health Organization’s Ordinal Scale for Clinical Improvement; *: defined as OSCI score for COVID-19 in acute phase ≥5; ^†^: serum albumin concentration <30 g/L; ^‡^: 6 missing data; ^||^: quinolones, beta-lactams, sulfonamides, macrolides, lincosamides, aminoglycosides, among others; ^§^: beta2-adrenergic agonists, inhaled corticosteroids, antihistamines, among others.
